# Usefulness of Two Independent DNA and RNA Tissue-Based Multiplex Assays for the Routine Care of Advanced NSCLC Patients

**DOI:** 10.3390/cancers12051124

**Published:** 2020-04-30

**Authors:** Elba Marin, Cristina Teixido, Elena Carmona-Rocha, Roxana Reyes, Ainara Arcocha, Nuria Viñolas, MªCarmen Rodríguez-Mues, Carlos Cabrera, Marcelo Sánchez, Ivan Vollmer, Sergi Castillo, Silvia Muñoz, Ivana G. Sullivan, Adela Rodriguez, Mireia Garcia, Silvia Alos, Pedro Jares, Antonio Martinez, Aleix Prat, Miguel Ángel Molina-Vila, Noemi Reguart

**Affiliations:** 1 Division of Medical Oncology, Hospital Clínic, 08036 Barcelona, Spain; ELMARIN@clinic.cat (E.M.); elecr6@gmail.com (E.C.-R.); rmreyes@clinic.cat (R.R.); aarcocha@clinic.cat (A.A.); nvinolas@clinic.cat (N.V.); mrodrigm@clinic.cat (M.R.-M.); adrodriguez2@clinic.cat (A.R.); alprat@clinic.cat (A.P.); 2 Translational Genomics and Targeted Therapeutics in Solid Tumors, Institut d’Investigacions Biomèdiques August Pi I Sunyer, 08036 Barcelona, Spain; teixido@clinic.cat; 3 Unitat Funcional de Tumors Toràcics, Hospital Clínic, 08036 Barcelona, Spain; msanche@clinic.cat (M.S.); vollmer@clinic.cat (I.V.); 4 Division of Pathology, Hospital Clínic, 08036 Barcelona, Spain; garcia01@clinic.cat (M.G.); salos@clinic.cat (S.A.); pjares@clinic.cat (P.J.); antonmar@clinic.cat (A.M.); 5 Division of Medical Oncology, Instituto Oncologico Dr. Rosell, Teknon Hospital, 08028 Barcelona, Spain; ccabrera@oncorosell.com; 6 Division of Thoracic Radiology, Hospital Clínic Barcelona, 08036 Barcelona, Spain; 7 Division of Medical Oncology, Hospital General de Granollers, 08402 Barcelona, Spain; sergimed77@gmail.com (S.C.); smunoz@fphag.org (S.M.); 8 Division of Medical Oncology, Hospital de la Santa Creu i Sant Pau, 08041 Barcelona, Spain; ISullivan@santpau.cat; 9 Molecular Biology Core Facility, Hospital Clínic, 08036 Barcelona, Spain; 10 Department of Medicine, University of Barcelona, 08036 Barcelona, Spain; 11 Laboratory of Oncology, Pangaea Oncology, Quirón Dexeus University Hospital, 08028 Barcelona, Spain; mamolina@panoncology.com

**Keywords:** advanced non-small cell lung cancer, molecular diagnostics, oncogenic drivers, mutations, targeted therapies

## Abstract

Personalized medicine is nowadays a paradigm in lung cancer management, offering important benefits to patients. This study aimed to test the feasibility and utility of embedding two multiplexed genomic platforms as the routine workup of advanced non-squamous non-small cell lung cancer (NSCLC) patients. Two parallel multiplexed approaches were performed based on DNA sequencing and direct digital detection of RNA with nCounter^®^ technology to evaluate gene mutations and fusions. The results were used to guide genotype-directed therapies and patient outcomes were collected. A total of 224 advanced non-squamous NSCLC patients were prospectively included in the study. Overall, 85% of samples were successfully characterized at DNA and RNA levels and oncogenic drivers were found in 68% of patients, with *KRAS*, *EGFR*, *MET*Δex14, *BRAF*, and *ALK* being the most frequent (31%, 19%, 5%, 4%, and 4%, respectively). Among all patients with complete genotyping results and follow-up data (*n* = 156), the median overall survival (OS) was 1.90 years (confidence interval (CI) 95% 1.69–2.10) for individuals harbouring an actionable driver treated with a matched therapy, compared with 0.59 years (CI 95% 0.39–0.79) in those not eligible for any targeted therapy and 0.61 years (CI 95% 0.12–1.10) in patients with no drivers identified (*p* < 0.001). Integrating DNA and RNA multiplexing technologies into the routine molecular testing of advanced NSCLC patients is feasible and useful and highlights the necessity of widespread integrating comprehensive molecular diagnosis into lung cancer care.

## 1. Introduction

The emergence of targeted therapy has revolutionized the management of patients with lung cancer by incorporating tumour molecular analyses into the routine diagnostic and therapeutic process [1]. At this time, non-small cell lung cancer (NSCLC) has undergone a paradigm shift from a purely histological to a molecular-based treatment approach, allowing for the selection of patients that would most benefit from targeted therapies, and thus impacting lung cancer care overall.

Adenocarcinoma is probably the lung cancer subtype that has most benefited from this molecular-based strategy, with an estimated frequency of oncogenic drivers higher than 50% [2]. About 25%–30% of these patients can benefit from a targeted therapy that has already been approved, and another 25%–30% are able to enroll in clinical trials. In order to personalize treatment decisions, several guidelines currently endorse routine genetic testing of four leading oncogenic drivers—*EGFR*, *ALK*, *ROS1*, and *BRAF*—in newly metastatic non-squamous NSCLC patients [3,4,5,6,7], while a number of other emerging molecular targets, such as *RET* and *NTRK* gene rearrangements, *MET* exon 14 skipping mutations (*MET*Δex14), and activating *HER2* mutations, are likely approaching clinical practice [8]. However, real-world data indicate that comprehensive biomarker testing is still not universal and remains suboptimal in daily clinical practice [8,9]. This is the case of Europeans, who face extreme inequality in access to appropriate molecular diagnosis, even within the same country, with huge differences in the national policies for reimbursement [10].

Our understanding of the molecular events that drive lung cancer pathogenesis has come from the progress made in highly efficient next-generation sequencing (NGS) technologies, which allow for a blanket testing of multiple potentially actionable driver genes from a single sample [11]. Several groups have already demonstrated the practicality of routine genetic testing in large cohorts of patients nationwide and the potential use of this information to guide treatment decisions [4,12,13,14,15,16,17,18,19,20,21,22]. However, the vast majority of those groups used DNA workflows to identify somatic variants—deletions, insertions, inversions, and substitutions—present in selected regions, whereas RNA-based multiplex analysis, which enables testing of several rearrangements and fusion partners, is still not in such common use [8,14] (Table S1).

In a previous study, we retrospectively validated a multiplexed RNA-based nCounter codeset for the detection of *ALK*, *ROS1*, and *RET* fusion transcripts in formalin-fixed and paraffin embedded (FFPE) samples from patients with advanced NSCLC and proved its advantage compared with standard diagnostic assays—immunohistochemistry (IHC) and fluorescent in situ hybridization (FISH) [23].

Here, we aimed to prospectively demonstrate the feasibility and usefulness of embedding two DNA and RNA tissue-based multiplex technologies in the real-life context of advanced lung cancer patients. The co-primary objectives set for this work were to determine the frequency of oncogenic alterations in advanced non-squamous NSCLC patients from our community and to measure overall survival (OS) in major molecular subgroups (driver/no-driver). The secondary objectives were to describe the characteristics of genotyped patients (age, gender, Eastern Cooperative Oncology Group (ECOG) performance status, and smoking history) and compare the outcomes according to treatment strategy (targeted/no-targeted therapy).

## 2. Results

### 2.1. Patient Cohort and Clinical Data

**Figure 1:**
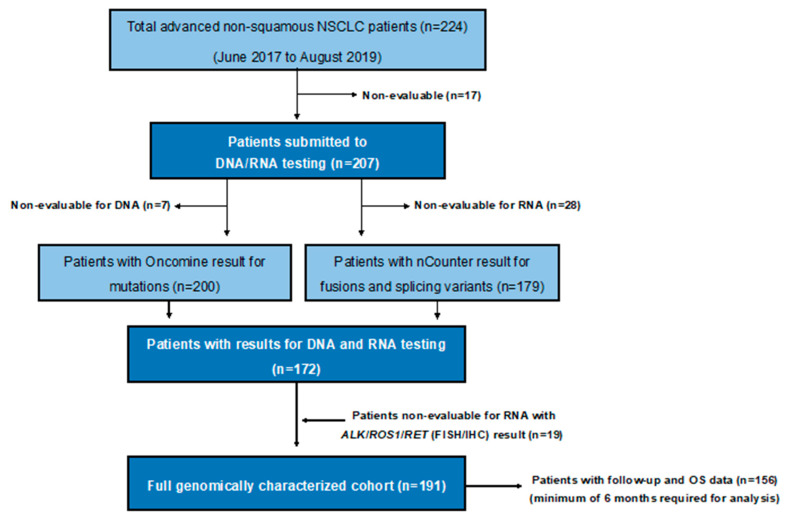
Flow diagram. NSCLC: non-small-cell lung cancer; OS: overall survival; FISH: fluorescence in situ hybridization; IHC: immunohistochemistry.

Between June 2017 and August 2019, a total of 224 advanced NSCLC patients were prospectively diagnosed at our institution. The last follow-up and update of the database was done in November 2019. Molecular DNA and/or RNA testing yielded an informative result in 207 patients (92%). Among them, 191 (85%) had both DNA and RNA informative results. All successfully genotyped patients had available clinical data and follow-up (Figure 1).

The primary characteristics of the patients are shown in Table 1. Median age was 70 years (interquartile range of 60–77), 63% of patients were men, and 57% had an ECOG performance status of 0 or 1 at metastatic disease diagnosis. Most patients (81%) were former or current smokers and 72% were diagnosed at advanced stage IV disease. The histology was adenocarcinoma for 92% of patients, while the remaining cases included not otherwise specified (NOS) or mixed NSCLC histologies. The majority of patients (84%) were treatment-naïve at the time of molecular assessment.

### 2.2. Molecular Characterization

**Figure 2:**
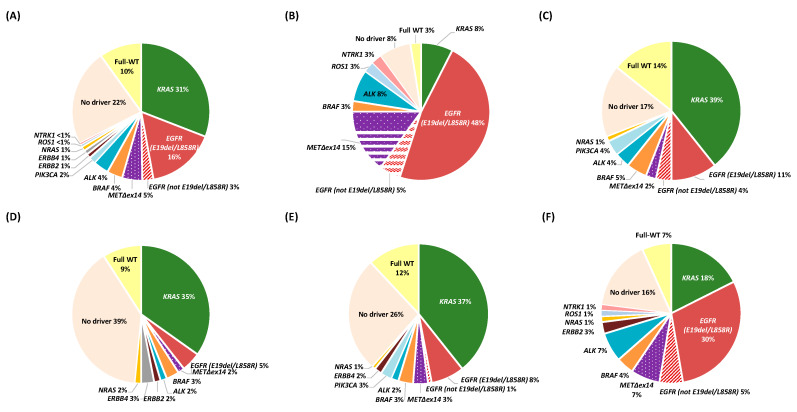
Genetic alterations identified. Pie charts depicting frequencies of driver alterations (*EGFR, BRAF, KRAS*, *MET*∆ex14, *ALK, ERBB2, ERBB4, PIK3CA, NTRK1, ROS1*, and *NRAS*) from 191 patients included in the prospective cohort (expressed as the percentage of positive samples for each molecular alteration relative to the total number of cases with an informative molecular result). Non-driver alterations include mutations at *AKT1, TP53, STK11, CTNNB1, SMAD4, DDR2, MAPK*, *MET*non∆ex14, *ALK* mutations, and cases with concomitant mutations (see Table 2 for further details). Wild-type (full-WT) tumours refer to those in which no genetic alteration was identified. (**A**) Overall population, (**B**) never smokers, (**C**) former smokers, (**D**) smokers, (**E**) male, and (**F**) female.

Among the 191 genotyped patients with a complete DNA and RNA molecular characterization, a genetic alteration was recorded in 90% of patients (Table 2). Driver alterations were found in 129 patients (68%), with *KRAS* (*n* = 59, 31%), *EGFR* (*n* = 36, 19%), *MET*Δex14 (*n* = 9, 5%), *BRAF* (*n* = 7, 4%), and *ALK* (*n* = 7, 4%) being the most commonly detected. Other less-common oncogenic driver genes identified were *ERBB4*, *ERBB2*, *PIK3CA*, *NRAS*, *NTRK1*, and *ROS1*. Non-driver alterations were identified in 43 patients (22%), whereas 19 full-wild-type (WT) patients (10%) did not harbour any alteration (Figure 2A).

**Figure 3:**
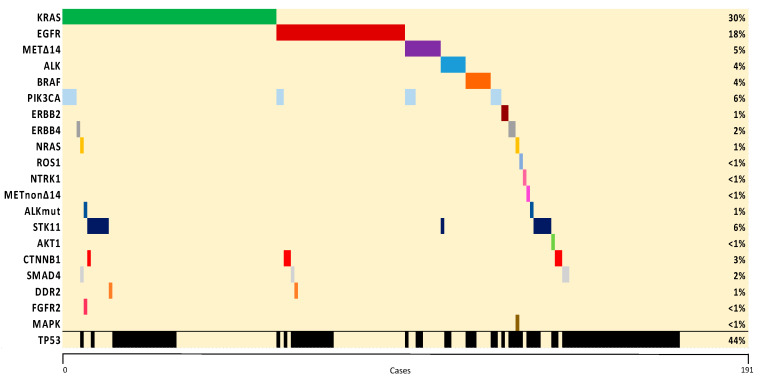
Co-mutation plot of genetic alterations identified. The percentage of samples with an alteration detected is noted on the right. Samples are displayed as columns and arranged to emphasize mutual exclusivity among mutations.

Sensitizing mutations (E19del and L858R) represented the most common *EGFR* subtype (86%), corresponding to 13% of all genotyped patients, whereas G12C was the most frequent *KRAS* variant identified (39%), accounting for 12% of all genotyped patients. Co-mutations were found in 51% of cases, with *TP53* (38%), *PIK3CA* (9%), and *STK11* (5%) being the most common. Co-mutations with *TP53* (32%) and *STK11* (10%) were the most common in *KRAS*-mutant, whereas *TP53* (39%) and *CTNNB1* (8%) were the most frequently described in *EGFR*-mutant patients. The most common oncogenic driver mutations were mutually exclusive except for *PIK3CA*. A co-mutation plot from the full sequencing of genotyped patients is shown in Figure 3.

The frequencies of the molecular alterations according to smoking habits and gender are shown in Figure 2B–F. Oncogenic drivers were more commonly found in women (77%), non-smokers (90%), and former smokers (69%), compared with men and smokers (61% and 51%, respectively). The frequency of *KRAS* mutation was consistently related to smoking exposure (8% vs. 39% vs. 35% in never-, former-, and current-smokers, respectively), while *EGFR* mutations were inversely related to smoking exposure (53% vs. 15% vs. 5%, respectively). In our series, two out of five women (35%) and two out of four non-smoking patients (53%) harboured an *EGFR* mutation. *MET*Δex14 mutations were more frequent in never-smokers (15% vs. 2% in patients with any smoking history). The subset of full-WT tumours (10%, *n* = 19) did not display differences between the different phenotypes studied, except for the non-smoking group, in which the percentage was slightly lower (3%).

The complete molecular results (DNA and RNA) were used to decide first-line treatments for most patients (172 out of 191, 90%), and 45 out of 191 patients (24%) were selected for targeted therapies (Table 2). Women, non-smokers, and ECOG 0–2 patients were significantly associated with targeted therapies, as well as the presence of *EGFR*, *MET*Δex14, *NTRK1*, *BRAF*, and *ALK* gene alterations.

### 2.3. Treatment Strategies and Overall Survival (OS)

Of the 36 patients carrying *EGFR* mutations, 27 (75%) were treated with a targeted agent, including first-generation (*n* = 14), second-generation (*n* = 8), and third-generation (*n* = 5) *EGFR* tyrosine-kinase inhibitors (TKIs). All patients with *ALK* rearrangements (*n* = 7) were treated with *ALK* inhibitors (four patients with crizotinib and three patients with new generation TKI). Three out of seven patients (43%) with *BRAF* mutations were treated with dabrafenib-trametinib (one *BRAF*-V600E and two non-V600E). Seven out of nine patients (78%) with the *MET*Δex14 alteration were treated with *MET* inhibitors (four in a clinical trial and three with crizotinib as compassionate use). The only patient diagnosed with an *NTRK1* gene rearrangement was treated with entrectinib in a compassionate use program.

**Figure 4:**
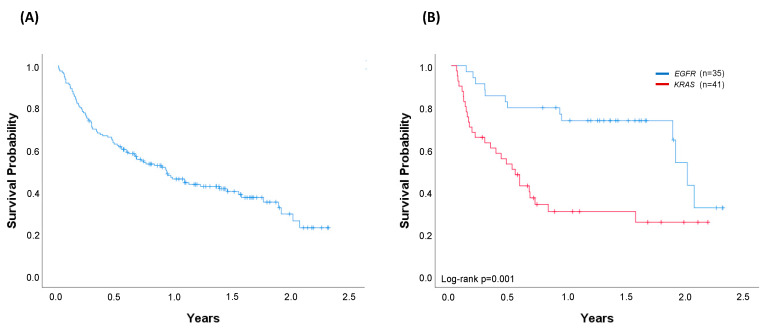
Overall survival (OS). (**A**) Entire cohort of successfully genotyped patients diagnosed with metastatic disease through April 2019 (*n* = 156). Median OS (95% CI): 0.93 years (0.63–1.24); (**B**) Patients harbouring *EGFR* (*n* = 35) or *KRAS* (*n* = 41) oncogenic driver mutations. For *KRAS*-mutated patients, 54% received chemotherapy, 12% immunotherapy, and 34% best supportive care. For *EGFR*-mutated patients, 77% received a matched therapy, 6% chemotherapy, and 17% best supportive care. Median OS (95%CI): *EGFR* 2.02 years (1.86–2.19); *KRAS* 0.55 years (0.36–0.74), log-rank *p* = 0.001. Vertical tick marks are censoring events.

**Figure 5:**
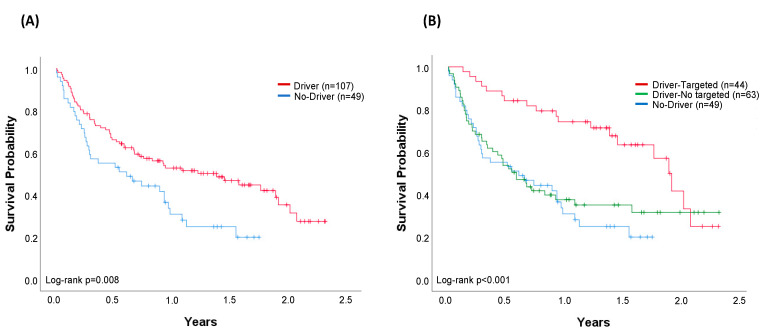
Comparison of overall survival (OS). (**A**) Cohort of patients (*n* = 156) with any oncogenic driver identified (*n* = 107) and patients with no driver (*n* = 49). Median OS (95% CI): driver, 1.40 years (0.75–2.02); no-driver, 0.61 years (0.12–1.10), log-rank *p* = 0.008. (**B**) Patients with no driver (*n* = 49) compared with patients with any oncogenic driver treated with targeted (*n* = 44) and patients with any oncogenic driver not treated with targeted therapy (*n* = 63). All patients in the driver-targeted group were treated with a genotype-directed therapy. No-driver and driver-no targeted patients were treated with chemotherapy (51% and 48%), immunotherapy (12% and 11%), and best supportive care (37% and 41%), respectively. Median OS (95% CI): driver-targeted 1.90 years (1.69–2.10); driver-no targeted 0.59 years (0.39–0.79); no driver 0.61 years (0.12–1.10), log-rank *p* < 0.001. Vertical tick marks are censoring events.

At the time of analysis, 87 patients were alive. The median OS of the whole cohort of 156 patients successfully evaluated through April 2019 was 0.93 years (95% confidence interval (CI) 0.63–1.24) (Figure 4A). Median OS was 1.40 years (95% CI 0.75–2.02) in the cohort of patients with an oncogenic driver identified (*n* = 107) and 0.61 years (95% CI 0.12–1.10) in the cohort of patients with no drivers identified (*n* = 49) (log-rank *p* = 0.008) (Figure 5A). Regarding the most common oncogenic drivers, median OS was significantly increased in *EGFR*-mutant patients (*n* = 35) compared with *KRAS*-mutant patients (*n* = 41) (median OS of 2.02 vs. 0.55 years, respectively, *p* = 0.001) (Figure 4B). Among *MET*Δex14, the third most common oncogenic driver alteration, the median OS was 1.77 years (95% CI 0.94–2.60).

When outcomes were compared in accordance with the treatment received (targeted/no-targeted), the median OS was 1.90 years (95% CI 1.69–2.10) in patients with a driver who received a matched genotype-directed therapy, compared with 0.59 years (95% CI 0.39–0.79) in patients with oncogenic drivers but not amenable to any targeted therapy (log-rank *p* = 0.002). Outcomes in this latter group are very similar to those from patients in whom no driver was identified (log-rank *p* = 0.559) (Figure 5B).

In the multivariable analysis, two variables were found to be associated with OS differences. Patients receiving targeted therapy had increased OS compared with those who were not amenable (hazard ratio (HR) 0.40, 95% CI 0.21–0.71, *p* = 0.002) and individuals with higher ECOG grades showed decreased OS values (HR 2.47, 95% CI 1.98–3.09, *p* < 0.001) (Table 3).

## 3. Discussion

Our study demonstrates the feasibility of routinely obtaining multiplex genomic and transcriptomic information useful to select therapies from patients with advanced non-squamous NSCLC. The successful implementation of molecular profiling of patients with lung cancer has already been reported (Table S1). However, outside the United States, genetic testing is variable and very little data on prospective genomic surveys in Europe and Spain have been reported. Our study is, to our knowledge, the first presentation of a full prospective real-world data genomic testing with related outcomes in a public healthcare hospital in Spain, and the first worldwide to integrate two parallel, multiplexed approaches based on DNA sequencing and direct digital detection of RNA with nCounter^®^ technology.

Using these two independent multiplexed DNA- and RNA-based platforms, we were able to screen for a total of 27 genes including tumour suppressor genes (TSG). This allowed us to provide a more precise characterization of the genetic alterations underlying non-squamous NSCLC, as compared with other studies in which data w only provided for ten [4] or six [13] of the most common or actionable driver genes (Table S1). In our study, we were able to identify oncogenic drivers in 68% of tumours, a percentage similar to the 64% recorded by The Lung Cancer Mutation Consortium (LCMC) in the USA [12], and higher than the 50% reported by the French Cooperative Thoracic Intergroup (IFCT) [13]. However, in our series, total gene alterations, comprising drivers and non-drivers, were identified in the vast majority of patients (90%). The broader molecular profiling gave us more precise information on the incidence of ‘real’ full-WT tumours, which comprised only 10% of the samples analysed, compared with the 15% previously reported [13].

Our screening failure rates were lower than previously reported, 7.6% in our series, compared with 26% and 35% in biopsies or cytologies for the LCMC [12], reinforcing their practicability in order to support individualised decisions in routine patient care. Although both techniques (NGS and nCounter) require minimum amounts of genetic material, our dropout percentage increased to 15% when considering those samples for which DNA or RNA testing could not be performed. Some groups, which also included combined DNA and RNA in their sequencing workflows, have reported lower invalid test rates of approximately 2%–3% [18,24]. However, these results do not correspond to the total screening failure rates, which should include samples with insufficient material, the major reason for failure in our series.

In our study, the prevalence of oncogenic and TSG (*TP53*, *STK11*, or *CTNNB1*) was aligned with the frequencies reported in other Caucasian cohorts (Table S2). Similarly, the highest incidence of drivers was found in never (90%) and former smokers (69%), endorsing the distinctive molecular signature of NSCLC according to smoking history.

Differences were observed for *EGFR* mutations, with an incidence (19%) significantly higher than the frequencies reported for other European and Spanish cohorts (11%–13%), but close to the series collected in the USA (23%) (Table S2). However, variations in mutation prevalence may exist depending on the variant filtering and annotation pipeline performed, and they are thus not entirely valid for comparisons. Whereas in some series, adenocarcinoma roughly accounted for 70% of the cases [13,17], in our cohort, we only included non-squamous NSCLC histologies, with a vast majority (90%) of adenocarcinomas. Moreover, some groups considered all tested patients for rating [13], including those partially genotyped, whereas we only considered those patients with a complete informative result. Finally, some studies only consider the most common *EGFR* sensitizing mutations (E19del and L858R), while in our cohort, we included all *EGFR* mutations in exons 18–21. Indeed, when considering only E19del and L858R *EGFR*-sensitizing mutations, our rates of EGFR positivity decrease to 16% (Figure 2).

Regarding the *MET* gene, we were unexpectedly able to identify 5% of patients carrying *MET*Δex14 skipping mutations, which represented the third most common driver alteration in our cohort. This percentage is over the 2%–3% usually reported [14,16,18,25,26] (Table S2), and endorses the advantage of using an RNA-based detection technique for *MET*Δex14 transcript identification [26]. Actually, commercially available DNA NGS panels have been reported to detect only 63% of literature-described *MET*Δex14 mutations [27]. Interestingly, contrary to other reports [3], our cohort of *MET*Δex14 patients was more frequently related to never-smokers (15%) and women (7%), highlighting the need for more data to properly characterize the phenotype related to this recently documented oncogenic driver.

In our cohort of genotyped patients, the median OS of 0.93 years was inferior to OS reported in previous studies [4]. However, our results reflect more accurately the real-world setting, and include a significant number of patients with ECOG 3–4 (24%) and not eligible for any treatment (16%).

The final goal of molecular testing is to personalise therapies and improve outcomes. Similar to previous reports [4,14], our molecular findings affected the treatment decision for 24% of the genotyped patients and 35% of patients with oncogenic drivers. Significant differences were observed when comparing median OS according to the presence or absence of a driver alteration (median OS 1.40 years vs. 0.61 years, respectively, log-rank *p* = 0.008). However, this survival advantage was not conditioned by the presence of a driver, but by the ability to receive a matched targeted therapy. Indeed, a significant increase in survival was obtained in patients with a driver who received a targeted therapy (1.90 years), while similar outcomes were observed in the cohort of oncogenic-driven patients not receiving any targeted therapy and those without drivers (median OS 0.59 years vs. 0.61 years, respectively) (Figure 5B). In the multivariable analysis, both treatment with targeted therapy and ECOG were independent variables of survival.

Remarkably, the majority (78%) of patients with the *MET*Δex14 received a targeted therapy with an *MET* inhibitor, achieving an outstanding median OS of 1.77 years and 12-month OS rate of 80%. These data compare favourably with the poor outcomes of this group of NSCLC patients [28,29], and emphasize the potential for targetability of this not-yet approved biomarker. Recently, the Food and Drug Administration (FDA) has granted a breakthrough therapy designation to capmatinib (INC280) as a first-line treatment for patients with *MET*Δex14 mutated NSCLC [30], based on the primary findings of the phase II GEOMETRY mono-1 study (NCT02414139) [31]. The final outcomes of this study are eagerly awaited, and we anticipate *MET*Δex14 to be soon added to the long list of actionable must-be-tested oncogenes in NSCLC.

The median OS in our *KRAS*-mutated cohort was dismal (0.55 years) and might be attributed to the lack of effective targeted therapies. Although this outcome clearly differs from the 2.41 years previously reported in the LCMC cohort [4], it resembles the OS recently reported in a retrospective analysis of chemotherapy-treated patients with advanced NSCLC and *KRAS* activating mutations (8.8 months vs. 13.5 months, *p* = 0.038, in *KRAS*-mutated and –WT, respectively) [32]. Although oncogenic *KRAS* could serve as an excellent drug target opportunity owing to its high incidence in lung cancer, its direct inhibition has proven to be challenging owing to a lack of traditional small molecule binding pockets on the protein [33]. However, emerging direct inhibitors of the most prevalent G12C variant—overall, the second most frequent driver-subtype alteration in our cohort accounting for 12% of all genotyped patients—have recently shown encouraging results [34], and further highlight the need for comprehensive panels to characterize lung cancer beyond the current biomarker recommendations.

Our single-institution prospective report may be subject to several inherent limitations. Because it was conducted with a rather small sample size, the findings provided on outcomes should be considered cautiously. Moreover, treatment was not randomly assigned, but decided by the physician, and a selection bias cannot be excluded. Finally, we did not adjust for several known confounding variables that might impact the outcomes, such as smoking status, stage at diagnosis, or prior therapies.

## 4. Materials and Methods 

### 4.1. Patients and Samples

Between June 2017 and August 2019, all of the patients at our institution with newly diagnosed advanced non-squamous NSCLC, as well as those for whom molecular profiling had not been previously completed, were prospectively included in our study with prior full informed patient consent and approval from the Local Ethical Committee (Comité Ético de Investigación Clínica del Hospital Clínic de Barcelona, V2-05/11/2016). The study was conducted in accordance with the principles of the Declaration of Helsinki. The decision to recommend a targeted therapy to a patient harbouring an actionable driver gene was left to the treating physician, who used the genomic data to select an ad-hoc therapy following clinical guidelines or via inclusion in a clinical trial.

Information on therapy and outcomes was collected regardless of whether the patient received targeted therapy. The data obtained were age; gender; histology; ECOG performance status; smoking history (never, former, or current smoker); TNM stage at diagnose, as defined by the seventh edition of the American Joint Committee on Cancer [35]; treatment setting at molecular assessment; the effect of the molecular results on the treatment decision with targeted therapy; and outcomes.

### 4.2. Molecular Analyses

All molecular analyses were performed on a routine basis at our institution using Oncomine^TM^ Solid Tumour DNA Kit and an in-house nCounter-based assay [23]. FFPE sample slides (4 µm) were stained with haematoxylin and eosin and the percentage of tumour cell content was estimated by a pathologist. Samples with tumour cell content higher than 20% were considered assessable for molecular analysis. Macrodissection was performed in those samples with a tumour content superior to 50%. In the case where only one technique could be performed, NGS was prioritized to nCounter.

Purified DNA (10 ng) was used as a template to generate genomic libraries using the Ion Torrent platform. The Oncomine^TM^ Solid Tumour DNA Kit (ThermoFisher Scientific, Life Technologies, CA, USA) interrogates somatic mutations on 22 genes, including *EGFR*, *ALK*, *ERBB2*, *ERBB*4, *FGFR1*, *FGFR2*, *FGFR3*, *MET*, *DDR2*, *KRAS*, *PIK3CA*, *BRAF*, *AKT1*, *PTEN*, *NRAS*, *MAP2K1*, *STK11*, *NOTCH1*, *CTNNB1*, *SMAD4*, *FBXW7*, and *TP53*. Libraries were sequenced using the Ion Personal Genome Machine platform (ThermoFisher Scientific) and analysed by the Ion Reporter Software (ThermoFisher Scientific) according to the manufacturer’s instructions.

For the nCounter assay (Nanostring Technologies, WA, USA), total RNA (25–200 ng) was directly hybridized with a customized codeset designed to detect specific fusion transcripts (7 *ALK*, 10 *ROS1*, 6 *RET*, and 2 *NTRK1*) and *MET*Δex14 mutations. All processes of hybridization, capture, clean-up, and digital data acquisition were performed as described in previous literature [23]. Regarding METΔex14 testing, log ratios were obtained dividing the normalized counts of the METΔex14 probe by the normalized counts for the MET WT probe. The cut-off for METΔex14 positivity was established as the average log ratio of the sample cohort plus two standard deviations.

Those samples without enough tumour content for a complete nCounter analysis were tested for *ALK*, *ROS1*, and *RET* fusions using alternative recommended tests. *ALK* status was checked by IHC (anti-ALK rabbit monoclonal antibody D5F3, Ventana, Tuzson, AZ) and FISH (ZytoLight^®^ SPEC ALK Dual Color Break Apart Probe). *ROS1* and *RET* were characterized by FISH (ZytoLight^®^ SPEC ROS1 Dual Color Break Apart Probe and ZytoLight^®^ SPEC RET Dual Color Break Apart Probe, respectively).

### 4.3. Molecular Subgroups

Molecular alterations—*EGFR*, *KRAS*, *NRAS*, *BRAF*, *MET*∆ex14, *ERBB2*, *ERBB4*, *PIK3CA*—and gene fusions—*ALK*, *ROS1*, *RET*, *NTRK1*—were referred to as oncogenic drivers [36,37,38]. Among the aforementioned, mutations at *EGFR*, *BRAF*, and *MET*∆ex14, and gene fusions at *ALK*, *ROS1*, *RET*, and *NTRK1* were considered actionable driver genes. Non-driver mutations included those reported in *TP53*, *STK11*, *CTNNB1*, *AKT1*, *SMAD4*, *DDR2*, *FGFR2*, *MAPK*, *ALK*, and *MET*non∆ex14. Tumours with an unchanged form were referred to as full WT tumours. Patients carrying co-mutations were considered once and categorized by the driver alteration.

### 4.4. Statistical Methods

Descriptive statistics, including the median and range for continuous variables and the percentages and frequencies for categorical variables, were tabulated and presented. OS was defined as the time from date of the metastatic disease diagnosis to the date of last follow-up or death. OS probability curves were calculated for all patients diagnosed with metastatic disease until April 2019 and for groups of interest by the Kaplan–Meier method, and compared with a two-sided log-rank test. For OS analysis, the cohort of patients with no oncogenic driver included those with non-driver mutations and full-WT tumours.

A *p*-value lower than 0.05 was considered for statistical significance. A multivariable Cox proportional hazard model was applied to adjust for potential confounders (clinical or molecular characteristics associated with OS). Adjusted HRs with 95% CI were calculated and the results were interpreted according to the following criteria: lack of association if HR = 1, increased risk of death if HR > 1, and lower risk of death if HR < 1. The SPSS v20.0 program was used for statistical analysis.

## 5. Conclusions

Our study demonstrates that embedding two DNA and RNA molecular genomic platforms early in the workup of patients with advanced NSCLC provides a complete understanding of the molecular NSCLC subsets that can be used to select patients who are candidates for targeted therapeutic intervention, resulting in significantly improved outcomes. The present paper echoes the need for expanding genomic surveys worldwide to integrate precision medicine into health care in order to guarantee the best outcomes for patients with advanced lung cancer.

## Figures and Tables

**Table 1:** Patient characteristics.

Variables	Total, *N*. (%) *(n* = 224)	Targeted Therapy, *N*. (%) (*n* = 45)	No Targeted Therapy, *N*. (%) (*n* = 179)
**Age at metastatic disease, median (IQR)**	70 (60–77)	70 (59–77)	69 (60–76)
**Sex**			
Women	82 (37)	30 (67)	52 (29)
Men	142 (63)	15 (33)	127 (71)
**Histology**			
Adenocarcinoma	206 (92)	44 (98)	162 (91)
Other ^1^	18 (8)	1 (2)	17 (9)
**Performance Status ^2^**			
0	32 (14)	11 (24)	21 (12)
1	96 (43)	23 (51)	73 (41)
2	40 (18)	7 (16)	33 (18)
3	48 (22)	4 (9)	44 (25)
4	5 (2)	0 (0)	5 (3)
Unknown	3 (1)	0 (0)	3 (1)
**Smoking history**			
Never	42 (19)	26 (58)	16 (9)
Former ^3^	99 (44)	15 (33)	84 (47)
Current	82 (37)	4 (9)	78 (43)
Unknown	1 (<1%)	0 (0)	1 (< 1)
**Stage at diagnose**			
I	8 (3)	2 (4)	6 (3)
II	22 (10)	3 (7)	19 (11)
III	33 (15)	6 (13)	27 (15)
IV	161 (72)	34 (76)	127 (71)
**Treatment setting at molecular diagnosis**			
Treatment naïve	188 (84)	35 (78)	153 (85)
Previously treated	36 (16)	10 (22)	26 (15)

IQR: interquartile range. ^1^ Other: includes not otherwise specified (NOS) or mixed non-small-cell lung cancer (NSCLC) histologies. ^2^ The ECOG (Eastern Cooperative Oncology Group) performance status of 0 (fully active); 1 (restricted in physically strenuous activity, but ambulatory and able to carry out work of a light or sedentary nature); 2 (ambulatory and capable of all self-care, but unable to carry out any work activities; up and about more than 50% of waking hours); 3 (capable of only limited self-care; confined to bed or chair more than 50% of waking hours); and 4 (completely disabled). ^3^ Patients who have not smoked for the last year or more.

**Table 2:** Genes with mutational or structural changes identified and targeted treatments received for patients with full genotyping (*n* = 191).

Molecular Alteration	Genotyping, *N*. (%) ^1^ (*n* = 191)	Targeted therapy, *N*. (%) ^2^ (*n* = 45)
**Driver oncogene ^3^**	**129 (68)**	**45 (35)**
*EGFR*	36 (19)	27 (75)
*EGFR E21*	1 (<1)	0 (0)
*EGFR E19del*	25 (13)	22 (88)
*EGFR E20*	4 (2)	1 (25)
*EGFR L858R*	6 (3)	5 (83)
*BRAF*	7 (4)	3 (43)
*BRAF V600E*	2 (1)	1 (50)
*BRAF non-V600E*	5 (3)	2 (40)
*KRAS*	59 (31)	0 (0)
*KRAS G12C*	23 (12)	0 (0)
*KRAS G12V*	14 (7)	0 (0)
*KRAS G12 (various)*	17 (9)	0 (0)
*KRAS G13*	2 (1)	0 (0)
*KRAS (other)*	3 (2)	0 (0)
*MET*∆ex14	9 (5)	7 (78)
*ALK*	7 (4)	7 (100)
*ERBB2 (HER2)*	2 (1)	0 (0)
*ERBB4*	2 (1)	0 (0)
*PIK3CA*	3 (2)	0 (0)
*NTRK1*	1 (<1)	1 (100)
*ROS1*	1 (<1)	0 (0)
*NRAS*	2 (1)	0 (0)
**Non-Driver oncogene ^4^**	**43 (22)**	**0 (0)**
*TP53*	31 (16)	0 (0)
*STK11*	3 (2)	0 (0)
*CTNNB1*	1 (<1)	0 (0)
>1 gene alteration	8 (4)	0 (0)
**Full-WT**	**19 (10)**	**0 (0)**

WT: wild-type ^1^ For percentages in this column, only patients with an evaluable molecular test are being considered, thus 191 is used as the denominator instead of the total 224 analysed. ^2^ Percentage among cases with detected mutation. ^3^ Genes considered driver oncogenes include *EGFR*, *BRAF*, *KRAS*, *MET*∆ex14, *ALK*, *ERBB2*, *ERBB4*, *PIK3CA*, *ROS1*, *NTRK1*, and *NRAS*. ^4^ Non-driver alterations included mutations at *TP53*, *STK11*, *CTNNB1*, *AKT1*, *SMAD4*, *DDR2*, *FGFR2*, *MAPK*, *ALK* mutations, and *MET*non∆ex14.

**Table 3:** Multivariable Cox proportional hazard model for survival, accounting for confounding variables in patients with full genotyping and diagnosed with metastatic disease through April 2019 (*n* = 156).

Variable	Multivariable		
	HR	95% CI	*p*
Age	1.00	0.98–1.02	0.851
Gender	0.98	0.61–1.60	0.951
ECOG	2.47	1.98–3.09	<0.001
Targeted Therapy	0.40	0.21–0.71	0.002

ECOG (Eastern Cooperative Oncology Group): performance status; HR: hazard ratio; CI: confidence interval.

## Supplementary Materials

The following are available online at https://www.mdpi.com/2072-6694/12/5/1124/s1, Table S1: Summary of comprehensive genotyping studies in NSCLC; Table S2: Summary of the Incidence of Driver Mutations in NSCLC.

## References

[1] Hunter D.J. (2016). Uncertainty in the era of precision medicine. N. Engl. J. Med..

[2] Pao W., Girard N. (2011). New driver mutations in non-small-cell lung cancer. Lancet Oncol..

[3] Planchard D., Hellmann M.D., Peters S., Popat S., Kerr K., Novello S., Smit E.F., Faivre-Finn C., Mok T.S., Reck M. (2018). Metastatic non-small cell lung cancer: ESMO Clinical Practice Guidelines for diagnosis, treatment and follow-up. Ann. Oncol..

[4] Kris M.G., Johnson B.E., Berry L.D., Kwiatkowski D.J., Iafrate A.J., Wistuba I.I., Varella-Garcia M., Franklin W.A., Aronson S.L., Su P.-F. (2014). Using multiplexed assays of oncogenic drivers in lung cancers to select targeted drugs. JAMA.

[5] Majem M., Juan O., Insa A., Reguart N., Trigo J.M., Carcereny E., García-Campelo R., García Y., Guirado M., Provencio M. (2019). SEOM clinical guidelines for the treatment of non-small cell lung cancer (2018). Clin. Transl. Oncol..

[6] Ettinger D.S., Aisner D.L., Wood D.E., Akerley W., Bauman J., Chang J.Y., Chirieac L.R., D’Amico T.A., Dilling T.J., Dobelbower M. (2018). NCCN guidelines insights: Non-Small cell lung cancer, version 5.2018. J. Natl. Compr. Cancer Netw..

[7] Kalemkerian G.P., Narula N., Kennedy E.B., Biermann W.A., Donington J., Leighl N.B., Lew M., Pantelas J., Ramalingam S.S., Reck M. (2018). Molecular testing guideline for the selection of patients with lung cancer for treatment with targeted tyrosine kinase inhibitors: American Society of Clinical Oncology endorsement of the College of American Pathologists/International Association for the study of lung cancer/Association for Molecular Pathology Clinical Practice guideline update. J. Clin. Oncol..

[8] Pennell N.A., Arcila M.E., Gandara D.R., West H. (2019). Biomarker testing for patients with advanced non–small cell lung cancer: Real-world issues and tough choices. Am. Soc. Clin. Oncol. Educ. Book.

[9] Remon J., Lacroix L., Jovelet C., Caramella C., Howarth K., Plagnol V., Rosenfeld N., Morris C., Mezquita L., Pannet C. (2019). Real-World utility of an amplicon-based next-generation sequencing liquid biopsy for broad molecular profiling in patients with advanced non-small-cell lung cancer. JCO Precis. Oncol..

[10] Europe LuCE (2017). LuCE Report on Lung Cancer. Disparities in Diagnosis, Care and Treatment Access. https://www.lungcancereurope.eu/2017/11/07/disparities-in-diagnosis-care-and-500treatment-access/.

[11] Hiley C.T., Le Quesne J., Santis G., Sharpe R., de Castro D.G., Middleton G., Swanton C. (2016). Challenges in molecular testing in non-small-cell lung cancer patients with advanced disease. Lancet.

[12] Sholl L.M., Aisner D.L., Varella-Garcia M., Berry L.D., Dias-Santagata D., Wistuba I.I., Chen H., Fujimoto J., Kugler K., Franklin W.A. (2015). Multi-Institutional oncogenic driver mutation analysis in lung adenocarcinoma: The lung cancer mutation consortium experience. J. Thorac. Oncol..

[13] Barlesi F., Mazieres J., Merlio J.-P., Debieuvre D., Mosser J., Lena H., Ouafik L.H., Besse B., Rouquette I., Westeel V. (2016). Routine molecular profiling of patients with advanced non-small-cell lung cancer: Results of a 1-year nationwide programme of the French Cooperative Thoracic Intergroup (IFCT). Lancet.

[14] Volckmar A.L., Leichsenring J., Kirchner M., Christopoulos P., Neumann O., Budczies J., Morais de Oliveira C.M., Rempel E., Buchhalter I., Brandt R. (2019). Combined targeted DNA and RNA sequencing of advanced NSCLC in routine molecular diagnostics: Analysis of the first 3000 Heidelberg cases. Int. J. Cancer.

[15] Li S., Li L., Zhu Y., Huang C., Qin Y., Liu H., Ren-Heidenreich L., Shi B., Ren H., Chu X. (2014). Coexistence of EGFR with KRAS, or BRAF, or PIK3CA somatic mutations in lung cancer: A comprehensive mutation profiling from 5125 Chinese cohorts. Br. J. Cancer.

[16] Suh J.H., Johnson A., Albacker L., Wang K., Chmielecki J., Frampton G., Gay L., Elvin J.A., Vergilio J.-A., Ali S. (2016). Comprehensive genomic profiling facilitates implementation of the national comprehensive cancer network guidelines for lung cancer biomarker testing and identifies patients who may benefit from enrollment in mechanism-driven clinical trials. Oncologist.

[17] Chatziandreou I., Tsioli P., Sakellariou S., Mourkioti I., Giannopoulou I., Levidou G., Korkolopoulou P., Patsouris E., Saetta A.A. (2015). Comprehensive molecular analysis of NSCLC: Clinicopathological Associations. PLoS ONE.

[18] Tsoulos N., Papadopoulou E., Metaxa-Mariatou V., Tsaousis G., Efstathiadou C., Tounta G., Scapeti A., Bourkoula E., Zarogoulidis P., Pentheroudakis G. (2017). Tumor molecular profiling of NSCLC patients using next generation sequencing. Oncol. Rep..

[19] Martín Martorell P., Huerta M., Compañ Quilis A., Abellán R., Seda E., Blesa S., Chaves F.J., Dualde Beltrán D., Roselló Keränen S., Franco J. (2017). Coexistence of EGFR, KRAS, BRAF, and PIK3CA mutations and ALK rearrangement in a comprehensive cohort of 326 consecutive Spanish nonsquamous NSCLC patients. Clin. Lung Cancer.

[20] Serizawa M., Koh Y., Kenmotsu H., Isaka M., Murakami H., Akamatsu H., Mori K., Abe M., Hayashi I., Taira T. (2014). Assessment of mutational profile of Japanese lung adenocarcinoma patients by multitarget assays: A prospective, single-institute study. Cancer.

[21] Bast E., Morrissey L., Tammireddy S., Shaw A.T., Borger D.R., Lennes I.T., Baselga J., Engelman J.A., Temel J.S., Sequist L.V. (2011). Implementing multiplexed genotyping of non-small-cell lung cancers into routine clinical practice. Ann. Oncol..

[22] Scheffler M., Ihle M.A., Hein R., Merkelbach-Bruse S., Scheel A.H., Siemanowski J., Brägelmann J., Kron A., Abedpour N., Ueckeroth F. (2019). K-ras mutation subtypes in NSCLC and associated co-occuring mutations in other oncogenic pathways. J. Thorac. Oncol..

[23] Reguart N., Teixidó C., Giménez-Capitán A., Paré L., Galván P., Viteri S., Rodríguez S., Peg V., Aldeguer E., Viñolas N. (2017). Identification of *ALK*, *ROS1*, and *RET* fusions by a multiplexed mRNA-based assay in formalin-fixed, paraffin-embedded samples from advanced non-small-cell lung cancer patients. Clin. Chem..

[24] Simarro J., Murria R., Pérez-Simó G., Llop M., Mancheño N., Ramos D., de Juan I., Barragán E., Laiz B., Cases E. (2019). Development, implementation and assessment of molecular diagnostics by next generation sequencing in personalized treatment of cancer: Experience of a public reference healthcare hospital. Cancers.

[25] Lu X., Peled N., Greer J., Wu W., Choi P., Berger A.H., Wong S., Jen K.Y., Seo Y., Hann B. (2017). Exon 14 mutation encodes an actionable therapeutic target in lung adenocarcinoma. Cancer Res..

[26] Reungwetwattana T., Liang Y., Zhu V., Ou S.-H.I. (2017). The race to target MET exon 14 skipping alterations in non-small cell lung cancer: The why, the how, the who, the unknown, and the inevitable. Lung Cancer.

[27] Poirot B., Doucet L., Benhenda S., Champ J., Meignin V., Lehmann-Che J. (2017). MET exon 14 alterations and new resistance mutations to tyrosine kinase inhibitors: Risk of inadequate detection with current amplicon-based NGS panels. J. Thorac. Oncol..

[28] Tong J.H., Yeung S.F., Chan A.W.H., Chung L.Y., Chau S.L., Lung R.W.M., Tong C.Y., Chow C., Tin E.K.Y., Yu Y.H. (2016). *MET* amplification and exon 14 splice site mutation define unique molecular subgroups of non-small cell lung carcinoma with poor prognosis. Clin. Cancer Res..

[29] Mazza V. (2019). New molecular drivers in NSCLC: The role of MET. Oncol. Res. Rev..

[30] Novartis Novartis Investigational Lung Cancer Therapy Capmatinib (INC280) Granted FDA Breakthrough Therapy Designation for Patients with MET-Mutated Advanced Non-Small Cell Lung Cancer (news release). https://www.novartis.com/news/media-releases/novartis-investigational-lung-cancer-therapy-capmatinib-inc280granted-fda-breakthrough-therapy-designation-patients-met-mutated-advanced-non-small-cell-lung.

[31] Wolf J., Seto T., Han J.-Y., Reguart N., Garon E.B., Groen H.J.M., Tan D.S.-W., Hida T., De Jonge M.J., Orlov S.V. (2019). Capmatinib (INC280) in METΔex14-mutated advanced non-small cell lung cancer (NSCLC): Efficacy data from the phase II GEOMETRY mono-1 study. J. Clin. Oncol..

[32] Hames M.L., Chen H., Iams W., Aston J., Lovly C.M., Horn L. (2016). Correlation between KRAS mutation status and response to chemotherapy in patients with advanced non-small cell lung cancer☆. Lung Cancer.

[33] Yang H., Liang S.-Q., Schmid R.A., Peng R.-W. (2019). New horizons in KRAS-mutant lung cancer: Dawn after darkness. Front. Oncol..

[34] Govindan R., Fakih M.G., Price T.J., Falchook G.S., Desai J., Kuo J.C., Strickler J.H., Krauss J.C., Li B.T., Denlinger C.S. (2019). 446PD phase I study of AMG 510, a novel molecule targeting KRAS G12C mutant solid tumours. Ann. Oncol..

[35] Goldstraw P., Crowley J., Chansky K., Giroux D.J., Groome P.A., Rami-Porta R., Postmus P.E., Rusch V., Sobin L. (2007). The IASLC lung cancer staging project: Proposals for the revision of the TNM stage groupings in the forthcoming (seventh) edition of the TNM classification of malignant tumours. J. Thorac. Oncol..

[36] Zhu Q.G., Zhang S.M., Ding X.X., He B., Zhang H.Q. (2017). Driver genes in non-small cell lung cancer: Characteristics, detection methods, and targeted therapies. Oncotarget.

[37] Mishra R., Hanker A.B., Garrett J.T. (2017). Genomic alterations of ERBB receptors in cancer: Clinical implications. Oncotarget.

[38] Ohashi K., Sequist L.V., Arcila M.E., Lovly C.M., Chen X., Rudin C.M., Moran T., Camidge D.R., Vnencak-Jones C.L., Berry L. (2013). Characteristics of lung cancers harboring NRAS mutations. Clin. Cancer Res. Off. J. Am. Assoc. Cancer Res..

