# Supplementary material for: Usefulness of Two Independent DNA and RNA Tissue-Based Multiplex Assays for the Routine Care of Advanced NSCLC Patients
Source: Cancers (Basel). 2020 Apr 30;12(5):1124. doi: 10.3390/cancers12051124 (PMC7281583; doi:10.3390/cancers12051124)
Supplement: Supplementary file 1 [file cancers-12-01124-s001.zip › cancers-778796-supplementary.pdf]

*Supplementary*

# Usefulness of Two Independent DNA and RNA Tissue-Based Multiplex Assays for the Routine Care of Advanced NSCLC Patients

**Elba Marin, Cristina Teixido, Elena Carmona-Roch, Roxana Reyes, Ainara Arcocha, Nuria Viñolas, MªCarmen Rodríguez-Mues, Carlos Cabrera, Marcelo Sánchez, Ivan Vollmer, Sergi Castillo, Silvia Muñoz, Ivana G. Sullivan, Adela Rodriguez, Mireia Garcia, Silvia Alos, Pedro Jares, Antonio Martinez, Aleix Prat, Miguel Ángel Molina-Vila and Noemi Reguart**

**Table S1: Summary of comprehensive genotyping studies in NSCLC.**

| Author | Continent | Population | Number of Patients (n) | Number Genes Tested (n) | NGSa (yes/no) | NGS Workflow (DNA and/or RNA) | Other Techniques (if not NGS) |
|---|---|---|---|---|---|---|---|
| Barlesi, F. et al. [13] (IFCT)a | Europe | Advanced NSCLC | 17664 | 6 genes | NO | - | • Pyrosequencing<br>• Fragment analysis assay<br>• SNaPshot<br>• Allele-specific PCR<br>• qPCR<br>• RT PCR<br>• Sanger<br>• HRM<br>• Mass array Sequenom<br>• Hybridization (Hybprobes)<br>• FISH |
| Suh, J. et al. [16] | America | NSCLC | 6832 | 236 genes + 19 genes' introns (Set 1)<br>or<br>315 genes + 28 genes' introns (Set 2) | YES<br>(hybrid-capture) | DNA | |
| Li, S. et al. [15] | Asia | NSCLC | 5125 | 4 genes | NO | - | • Multiplex PCR<br>• Allele-specific primer extensión<br>• Hybridization |
| Scheffler, M. et al. [22] | Europe | NSCLC | 4507 | 14 genes | YES<br>(amplicon-based) | DNA | |
| Volckmar, AL. et al. [14] (IPH)b | Europe | Advanced NSCLC | 3000 | 41 or 42 or 38 genes (Panels LCPv1/v2/v3) + 51 or 159 fusion transcripts (Panels LFPv1/v2) | YES<br>(amplicon-based) | DNA and RNA | |
| Sholl, LM. et al. [12]<br>Kris, MG. et al. [4] (LCMC)c | America | Lung ADC | 1542 | 10 genes | NO | - | • SNaPshot<br>• Mass spectrometry<br>• Sanger<br>• FISH |
| Chatziandreou, I. et al. [17] | Europe | NSCLC | 956 | 6 genes | NO | - | • HRM<br>• Pyrosequencing<br>• RT PCR |

| | | | | | | | |
|---|---|---|---|---|---|---|---|
| | | | | | | | • IHC<br>• FISH |
| Serizawa, M. et al. [20] | Asia | Lung ADC | 845 | 15 genes | NO | - | • Pyrosequencing<br>• Fragment analysis assay<br>• qPCR<br>• RT PCR<br>• IHC<br>• FISH |
| Bast, E. et al. [21] | America | NSCLC | 589 | 16 genes | NO | - | • SNaPshot<br>• FISH |
| Tsoulos, N. et al. [18] | Europe | NSCLC | 512 | 23 genes + 70 fusion transcripts (*ALK, RET, ROS1 and NTRK1*, Ion AmpliSeq RNA Fusion Lung Cancer Research Panel) | YES (amplicon-based) | DNA and RNA | |
| Martín-Martorell, P. et al. [19] | Europe | NSCLC | 326 | 5 genes | YES (amplicon-based; single gene) | DNA | |
| Simarro J, et al. [24] | Europe | NSCLC | 106 | 22 genes (oncomine-DNA) + 23 fusion transcripts (*ALK, RET, ROS1, NTRK1* oncomine RNA) | YES (amplicon-based) | DNA and RNA | |
| Marin E. et al. | Europe | Advanced non-squamous NSCLC | 191 | 22 genes (oncomine-DNA) + 25 fusion transcripts (*ALK, RET, ROS1 and NTRK1*, nCounter) + *MET*Δex14 (nCounter) | YES (amplicon-based and RNA-counting) | DNA and RNA | |

[a] French Cooperative Thoracic Intergroup (IFCT); [b] Institute of Pathology Heidelberg (IPH); [c] Lung Cancer Mutation Consortium (LCMC); NGS, Next Generation Sequencing; NSCLC, non-small cell lung cancer; ADC, Adenocarcinoma; IHC, Immunohistochemistry; FISH, Fluorescent in Situ Hybridization; HRM, High Resolution Melting; PCR, Polymerase Chain Reaction; RT PCR, Real Time PCR; qPCR, quantitative PCR.

**Table S2.** Summary of the Incidence of Driver Mutations in NSCLC.

| Author | Population | *KRAS* | *EGFR* | *MET*Δ*ex14* | *BRAF* | *ALK* | *PIK3CA* | *ERBB2* | *ERBB4* | *NRAS* | *ROS1* | *NTRK1* |
|---|---|---|---|---|---|---|---|---|---|---|---|---|
| Barlesi, F. et al. [IFCT] [a] [13] | Caucasian | 29 | 11 | - | 2 | 5 | 2 | 1 | - | - | - | - |
| Suh, J. et al. [16] | Caucasian | 32 | 20 | 3 | 6 | 4 | 5 | 6 | - | 2 | 1 | <1 |
| Li, S. et al. [15] | Asian | 8 | 36 | - | <1 | - | 3 | - | - | - | - | - |
| Volckmar, AL. et al. [14], (IPH) [b] | Caucasian | 34 | 18 | 2 | 5 | 3 | 6 | 2 | 2 | 2 | 1 | - |
| Sholl, LM. et al. [12] and Kris, MG. et al. [4], (LCMC) [c] | Caucasian | 25 | 23 | - | 2 | 8 | <1 | 3 | - | <1 | - | - |
| Chatziandreou, I. et al. [17] | Caucasian | 26 | 11 | - | 3 | 4 | 4 | - | - | - | - | - |
| Serizawa, M. et al. [20] | Asian | 9 | 35 | - | <1 | 5 | 3 | 2 | - | <1 | - | - |
| Bast, E. et al. [21] | Caucasian | 24 | 13 | - | 2 | 5 | 4 | <1 | - | 1 | - | - |
| Tsoulos, N. et al. [18] | Caucasian | 25 | 11 | 2 | 4 | 2 | 3 | 2 | 1 | <1 | <1 | 0 |
| Martín-Martorell, P. et al. [19] | Caucasian | 39 | 24 | - | 3 | 9 | 10 | - | - | - | - | - |
| Simarro P. et al. [24] | Caucasian | 28 | 13 | - | 3 | 5 | 4 | 3 | - | 1 | 1 | - |
| Marin, E. et al. | Caucasian | 31 | 19 | 5 | 4 | 4 | 2 | 1 | 1 | 1 | <1 | <1 |

[a] French Cooperative Thoracic Intergroup (IFCT); bInstitute of Pathology Heidelberg (IPH); cLung Cancer Mutation Consortium (LCMC); WT; wild type;
